# Learning pain from others: a systematic review and meta-analysis of studies on placebo hypoalgesia and nocebo hyperalgesia induced by observational learning

**DOI:** 10.1097/j.pain.0000000000002943

**Published:** 2023-06-15

**Authors:** Stefanie H. Meeuwis, Mateusz T. Wasylewski, Elżbieta A. Bajcar, Helena Bieniek, Wacław M. Adamczyk, Sofiia Honcharova, Marianna Di Nardo, Giuliana Mazzoni, Przemysław Bąbel

**Affiliations:** a Jagiellonian University, Institute of Psychology, Pain Research Group, Kraków, Poland; b The Jerzy Kukuczka Academy of Physical Education, Institute of Physiotherapy and Health Sciences, Katowice, Poland; c Department of Dynamic, Clinical Psychology and Health, Sapienza University of Rome, Rome, Italy

**Keywords:** Placebo hypoalgesia, Nocebo hyperalgesia, Placebo effects, Nocebo effects, Observational learning, Social learning

## Abstract

Observing someone experience pain relief or exacerbation after an intervention may induce placebo hypoalgesia or nocebo hyperalgesia. Understanding the factors that contribute to these effects could help in the development of strategies for optimizing treatment of chronic pain conditions. We systematically reviewed and meta-analyzed the literature on placebo hypoalgesia and nocebo hyperalgesia induced by observational learning (OL). A systematic literature search was conducted in the databases PubMed, PsycINFO, Web of Science, ScienceDirect, PsycARTICLES, Scopus, and Academic Search Ultimate. Twenty-one studies were included in the systematic review, 17 of which were suitable for meta-analysis (18 experiments; n = 764 healthy individuals). The primary end point was the standardized mean difference (SMD) for pain following placebo cues associated during OL with low vs high pain. Observational learning had a small-to-medium effect on pain ratings (SMD 0.44; 95% confidence interval [CI] 0.21-0.68; *P* < 0.01) and a large effect on pain expectancy (SMD 1.11; 95% CI 0.49-2.04; *P* < 0.01). The type of observation (in-person vs videotaped) modulated the magnitude of placebo hypoalgesia/nocebo hyperalgesia (*P* < 0.01), whereas placebo type did not (*P* = 0.23). Finally, OL was more effective when observers' empathic concern (but no other empathy-related factors) was higher (*r* = 0.14; 95% CI 0.01-0.27; *P* = 0.03). Overall, the meta-analysis demonstrates that OL can shape placebo hypoalgesia and nocebo hyperalgesia. More research is needed to identify predictors of these effects and to study them in clinical populations. In the future, OL could be an important tool to help maximize placebo hypoalgesia in clinical settings.

## Abstract 2

Supplemental Digital Content is Available in the Text.

## 1. Introduction

Placebo hypoalgesia is observed when a sham intervention (placebo) results in pain relief, whereas nocebo hyperalgesia manifests as increased pain following this type of intervention. Learning processes have been shown to be involved in shaping placebo hypoalgesia and nocebo hyperalgesia.^5,61^ Classical conditioning is the most extensively investigated learning process that produces both effects.^2^ According to the principles of classical conditioning, an initially inactive intervention, when associated with an intervention that produces changes in pain sensations, is able to produce similar results. Placebo hypoalgesia can also be acquired through operant conditioning.^1^ In this process, an inactive intervention effectively influences pain sensations, for instance, because its use in the past was contingently rewarded (ie, by the experience of less pain) or not punished (ie, by increased or continued pain). Verbal suggestions, ie, providing participants with information on possible changes in pain sensations after a sham intervention, are also effective in shaping placebo and nocebo effects. People are social beings and also learn indirectly, ie, by observing the experiences of other people.^9,10^ The observation of another person (a model) who demonstrates pain relief or pain exacerbation following a sham intervention may elicit similar responses in an observer, on whom the same placebo is subsequently used. Previous studies on classical conditioning and verbal suggestions have shown that although both processes have the potential to induce placebo and nocebo effects,^53,68^ the magnitude of placebo hypoalgesia and nocebo hyperalgesia could differ depending on the particular learning process.^24^ In the light of these data, it seems likely that the contribution of observational learning (OL) to each of these 2 effects may also be not equal; however, this has yet to be established because no study to date has systematically summarized and compared the available data on the efficacy of OL in shaping placebo hypoalgesia and nocebo hyperalgesia.

The placebo and nocebo effects that are elicited by OL and described in the existing literature vary in magnitude: some studies report large effects,^21^ whereas others report small to no effects.^6^ Potential factors that influence the efficacy of OL may be found, for instance, in between-study differences in the manner in which people observe the effects (eg, in-person, on video). The approach proposed by Bajcar and Bąbel^5^ integrates the various ways of observing pain and placebo-related information with the theoretical assumptions of the social learning theory.^9,10^ According to this approach, social information about pain can influence the experience of pain, for instance, through presentation of the pain ratings without the model being present (ie, verbal modelling) or through observation of the model themselves, which can take place either in person (direct behavioural modelling) or through videotapes or pictures (indirect behavioural modelling, or symbolic modelling). Verbal modelling as a type of observational learning is distinct from, for instance, verbal suggestions or instructions: the latter typically include direct and precise suggestions about the to-be-expected effects of a treatment on a person's own pain experience. On the contrary, for placebo effects to occur after verbal modelling, the observer has to actively process the pain ratings of others and translate them to what they themselves would expect (ie, in place of the observed person). Verbal modelling can occur, for instance, when written accounts of beneficial pain treatment outcomes are described on the internet and influence a person's own expectations about a similar treatment. Behavioural modelling can take place, eg, when other patients in pain are observed in the clinical setting, whereas symbolic modelling can occur, for instance, when people in pain are observed on videos on TV. Some work has directly compared in-person observation with videotaped observation,^38^ but overall, studies tend to use a single approach to observational learning. A systematic comparison across studies is needed to verify how differential methods can influence the magnitude of OL.

Theoretical accounts, including the approach proposed by Bajcar and Bąbel,^5^ emphasize the mediating role of expectancies in shaping placebo and nocebo effects.^13,22,41^ However, studies on classically conditioned placebo effects show that pain-related expectancies may not always be involved in placebo hypoalgesia^3,39^ or nocebo hyperalgesia.^3,18,39^ Moreover, in some cases, these expectancies—even when modified because of learning—are not predictive of placebo hypoalgesia.^3^ These data suggest that the involvement of conscious processes in placebo effects induced by OL may not be evident, but instead, that they may be part of the nondeclarative memory and reflect, for instance, priming processes. Summarizing the available data on the involvement of conscious expectancy in OL-induced placebo hypoalgesia and nocebo hyperalgesia may shed some light on the underlying processes.

According to Bajcar and Bąbel's approach,^5^ the effectiveness of OL in shaping placebo and nocebo effects may also depend on the observer's characteristics. In particular, empathy, defined as “a sense of knowing the experience of another person with cognitive, affective, and behavioural components,”^31^ may be crucial in this process. Neuroimaging studies have shown that neural structures activated in reaction to pain and associated with empathy for pain partially overlap.^45,59^ Variation in the impact of OL on expectancy and the influence of empathy may contribute to variance in placebo and nocebo effects, but none to date have systematically compared these factors across studies on observational learning–evoked placebo hypoalgesia and nocebo hyperalgesia.

In the past decade, experimental studies on placebo effects generated by OL have intensified. However, the data collected in these studies are not always conclusive and need to be reviewed to verify the assumptions of the theoretical approach that describes observationally induced placebo effects.^5^ The current article is the first to systematically review studies on the effects of social learning on placebo and nocebo effects. First, the literature was summarized, and next, a meta-analysis was conducted to answer the following questions using a stepwise approach (ie, in which, first, the effects of OL in general were assessed and, second, separated for placebo and nocebo effects): (1) is OL an effective method for modulating pain experience following an inert intervention?; (2) do placebo hypoalgesia and nocebo hyperalgesia induced by OL differ in magnitude?; (3) does how a model is presented (ie, in-person or videotaped) affect the magnitude of placebo hypoalgesia and nocebo hyperalgesia?; (4) are pain expectancies related to placebo hypoalgesia and nocebo hyperalgesia?; (5) is the empathy of the observer related to the magnitude of placebo hypoalgesia and nocebo hyperalgesia?

## 2. Methods

The review protocol was designed according to the Preferred Reporting Items for Systematic Review and Meta-Analyses Protocols (PRISMA-P checklist form) guidelines.^49^ It follows the recommendations for data searching and data processing described in the Cochrane Handbook for Systematic Reviews^36^ and was preregistered in the PROSPERO database (ID: CRD42021241091). All reviewers were trained in the screening and extraction procedures before conducting these stages. The review procedures were piloted to ensure the validity and consistency of the assessment strategies. The search and assessment criteria were discussed and standardized a priori.

### 2.1. Databases and search strategy

Seven databases were strategically searched for relevant studies from their inception until February 13, 2022: PubMed, PsycINFO, Web of Science, ScienceDirect, PsycARTICLES, Scopus, and Academic Search Ultimate. The search strategy combined keywords regarding observational and social learning with keywords related to placebo and nocebo effects or placebo hypoalgesia and nocebo hyperalgesia, respectively; all fields were searched (title, abstract, keywords etc). The search strategy used for the PubMed database is shown in Supplementary Table S1 (available at http://links.lww.com/PAIN/B844). Similar strategies were used for other databases and were modified where needed to suit the particular search engine. These modifications included shortening the strategy used in the ScienceDirect database (a maximum of 8 boolean operators may be used per field) and changing the strategy syntax in Scopus. We did not include specific keywords for population (eg, healthy, adult humans, pain patients) or outcomes (eg, pain response or behaviour). Terms for the comparator and study type were also omitted to avoid potential automatic exclusion of relevant studies. We did not use any automatic filters to restrict the search results. All the necessary exclusions were done manually by the reviewers.

The reference lists from relevant articles were screened. Furthermore, we also ran the included studies through the Connected Papers search engine to identify any additional articles.

### 2.2. Selection of relevant studies

Studies were included in the systematic review if (1) OL was used to induce placebo hypoalgesia or nocebo hyperalgesia; (2) self-reported pain was measured on a pain scale (eg, numeric rating scale or visual analogue scale); and (3) the study involved healthy, pain-free individuals or patients experiencing either chronic or acute pain. We included only articles published in English. If a study was too dissimilar to be meta-analyzed (eg, there was no pain stimulation comparable to those featured in the included studies or OL was reinforced by a conditioning procedure), but it still generally fulfilled the inclusion criteria, it was included in the systematic review but not in the meta-analysis. Review articles, meta-analyses, and conference communications were excluded; however, we screened the reference lists of reviews relevant to the topic for potential inclusions. There were no restrictions regarding the publication date. Detailed inclusion and exclusion criteria are provided in Supplementary Table S2 (available at http://links.lww.com/PAIN/B844).

First, the reviewers (M.W., E.B., S.M., H.B., S.H., M.N.) were grouped into independent pairs. Each pair assessed one-third of the titles and abstracts retrieved from the database searches for eligibility according to the inclusion/exclusion criteria. In the next stage, full texts were reviewed using the same criteria. Any disagreements between the 2 assessors were resolved by discussion with a third reviewer (P.B., G.M.). After including the studies identified through the initial database search, 3 reviewers (H.B., S.H., M.N.) independently searched for studies related to previously included articles using the Connected Papers^25^ search engine.

### 2.3. Risk of bias assessment and publication bias

The checklist developed by Downs and Black^26^ and recommended in the Cochrane Handbook for Systematic Reviews of Interventions^36^ was used to assess the potential risk of bias of the included studies. This checklist facilitates the assessment of bias in several areas, including reporting and internal and external validity (bias and confounding). The risk of bias was assessed independently by 2 reviewers (E.B., M.W.). Disagreements between the reviewers were resolved by discussion between them. For the purposes of our review, we modified the original Downs and Black checklist to better fit the specific type of the included studies, ie, basic placebo mechanism studies. The original Downs and Black checklist has a maximum of 24 points; after dropping four 1-point items, we were left with an assessment tool that yields a maximum 20-point score (details about the checklist modifications are provided in Supplementary Table S3, available at http://links.lww.com/PAIN/B844). For each item, studies were assigned points if there was evidence of bias minimization (eg, blinding participants and/or assessors, baseline characteristics sufficiently described etc). A study was given zero points if it used practices that may have introduced bias (eg, no blinding or the statistical tests used to assess the main outcomes were inappropriate, etc), or if bias minimization could not be determined. The lower the score on the scale, the higher the potential bias, with studies scoring 1 to 7 being considered high bias, 8 to 14 moderate bias, and 15 to 20 low bias. The risk of bias assessment sheet is available in Supplementary Table S4 (available at http://links.lww.com/PAIN/B845). To test for potential publication bias, a graphical funnel plot with the effect sizes for pain plotted against the magnitude of the standard error was inspected, and Egger's test was performed.

### 2.4. Data extraction

Sample demographics, details about the intervention, study design and outcomes, and data relevant to the meta-analysis were extracted from all the included studies by 2 independent reviewers (S.M., M.W.). These data included sample size, participants' age and sex, study design, the type of effect the researchers aimed to induce, details on how the model was presented, and the type of placebo, ie, with medical connotations (eg, a cream) or abstract nonmedical cues (eg, colors, geometric shapes). We additionally extracted information about the nature of noxious stimuli (electrocutaneous, thermal or pressure), assessment tools for pain, expectancy, and empathy, along with mean ratings in the experimental and control groups for these outcomes. In addition, information on statistical associations (eg, Pearson coefficients) between placebo hypoalgesia/nocebo hyperalgesia, and empathy was extracted, as were data on other study outcomes, including pain unpleasantness, physiological outcomes (eg, skin conductance or heart rate). If relevant, supplementary appendices of the included studies were checked for additional information. When data were missing, we contacted the corresponding author of the publication to obtain the missing results. If we received no reply or the data could not be obtained within 2 weeks, we proceeded with our analysis, relying only on the information that was already available to us. The results obtained by each reviewer were compared, and any disagreements were resolved by discussion. The extraction sheet containing the raw data used for the analyses is available in Supplementary Table S5 (available at http://links.lww.com/PAIN/B845).

### 2.5. Statistical analysis

Before conducting the meta-analysis, the numerical data from the included studies regarding participant characteristics as well as pain, expectancy, and empathy outcomes were standardized (eg, standard errors of the mean were converted to standard deviation by multiplying the former by the square root number of evaluated participants). The summary score for the magnitudes of placebo and nocebo effects in pain was based on the difference between pain ratings for stimuli that were associated with low pain (low-pain cues, eg, orange color) and high pain (high-pain cues, eg, blue color) through observational learning. Difference scores were calculated by subtracting the low cue pain rating from the high cue rating (Δ |H-L|, ie, nocebo − control, or control − placebo). The SDs for the difference scores were obtained with the formula √(SD1^2^ + SD2^2^ − [2·r·SD1·SD2]), with correlations between ratings set at 0.5. If a study comprised multiple OL groups, mean pain ratings across groups and pooled SDs were calculated across OL groups, weighted for the number of participants within each group. Then, summary scores for the magnitude of placebo and nocebo effects were calculated. If more than 2 groups were combined, pooling was done sequentially (ie, groups 1 and 2 were combined first, then the pooled group was combined with group 3 etc). Similar calculations were used for the other outcomes. When mean values and the dispersion measure were reported separately for different subgroups (eg, mean age was reported for experimental and control groups separately), the groups were also pooled.

The meta-analyses were based on the random-effect model with within-groups comparison of the standardized mean difference (SMD) between high-pain–associated cues (either nocebo or control, depending on the study) and low-pain–associated cues (placebo or control) for pain intensity. A positive SMD indicated a bigger magnitude of the effect of OL on either pain or expectancy, regardless of direction (pain decrease or increase). The pooled effect was weighted by the sample size in the given study and estimated with 95% confidence intervals (CIs). The primary analysis was conducted on the difference in pain ratings (ie, the magnitude of placebo/nocebo effects; primary end point). As secondary end points, the efficacy of OL for pain was compared for the (1) method of observation (behavioural modelling vs symbolic and verbal modelling) and (2) the nature of the placebo used for the manipulation (medical vs nonmedical). Other secondary outcomes included meta-analysis of the effects of OL on expectancy ratings across the studies using identical methods as for pain ratings. Meta regression was used to assess the relationship between the magnitude of placebo/nocebo effects and empathy scores. Correlation coefficients were pooled after Fisher z-transformation; heterogeneity between studies was assessed using the I^2^ statistic. The random effects model correlations were then calculated using the inverse variance method. All analyses were performed using R Studio (v. 2022.07.1) and the “meta”^8^ and “metacor”^44^ packages.

Originally, we aimed to separately analyze (1) the efficacy of OL in pain, (2) the method of observation, (3) the nature of the placebo used, and (4) the effects of OL on expectancy and pain for studies that involved inducing either the placebo or the nocebo effect according to the authors. Upon close scrutiny of the experimental manipulation used to induce the placebo or nocebo effect in each study included in the meta-analysis, we came to the conclusion that studies labelled as “placebo” and “nocebo” studies by their authors often featured similar if not identical pain manipulations (ie, pain cues rated as “high” vs “low”; see the Discussion section for details). Therefore, the analyses that featured this division are interpreted very cautiously. Moreover, in the results, we present the placebo/nocebo subgroup analysis only for the main analysis (ie, the effects of OL on pain ratings). The other secondary analyses that featured this subdivision are presented in Supplementary Appendix 1 (available at http://links.lww.com/PAIN/B844). In addition, we were unable to fully analyze the correlation between pain expectancy and the incidence of pain modulation through OL because most studies included in the analysis did not report correlational data between expectancy and pain ratings. We were only able to conduct an exploratory meta-analysis of OL on expectancy.

## 3. Results

### 3.1. Search strategy

**Figure 1.**
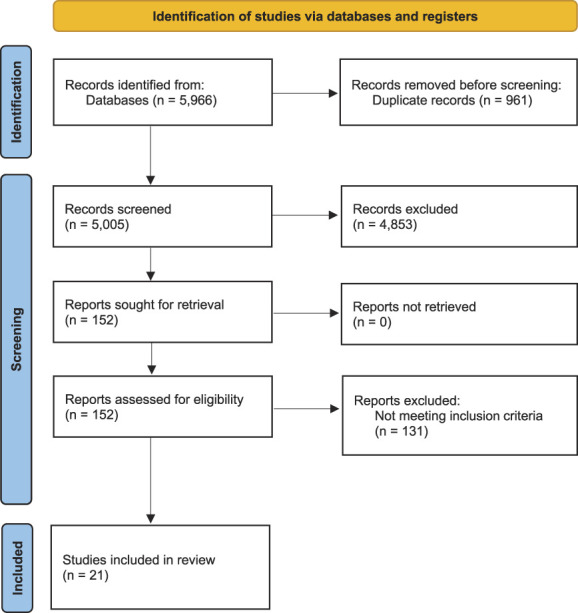
PRISMA flow diagram^32^ (modified). k, number of records; PRISMA, preferred reporting items for systematic review and meta-analyses.

The database search yielded 5966 records in total. After duplicates were removed, 5005 unique titles and abstracts were screened. This first step of the systematic search resulted in 152 full-text articles that were screened for eligibility. Of them, 21 studies were included in the systematic review and 17 studies (describing 18 discrete experiments) were considered suitable for pooling in the meta-analysis (Fig. 1).

### 3.2. Systematic review

#### 3.2.1. Study characteristics

The study characteristics are shown in Table 1. In total, 1629 participants were enrolled in the studies. Most studies (63.64%, k = 14) were of a randomized parallel-group design. The majority (90.91%, k = 20) did not involve any form of baseline pain assessment before the placebo or nocebo effect induction by OL.

**Table 1:** Characteristics of included studies.

Characteristic	Category	k (%)
	No. of articles included in the review	21
	No. of experiments	22 (100.00)
	Total number of evaluated participants	1375 (100.00)
	% Males	26.76
Study design	Cross-over	1 (4.55)
	Parallel design	14 (63.64)
	Within-subject	7 (31.81)
Was there any form of baseline pain assessment?	Yes	2 (9.09)
	No	20 (90.91)
Type of effect aimed to be induced by OL (as specified by the authors of the study)	Placebo	10 (45.45)
	Nocebo	9 (40.91)
	Both	3 (13.64)
The way the model was presented	Direct	6 (27.27)
	Indirect	15 (68.18)
	Both*	1 (4.55)
Sex of the model	Male	6 (27.27)
	Female	11 (50.00)
	Both	5 (22.73)
Type of placebo	Medical	10 (45.45)
	Non-medical	12 (54.55)
Nature of noxious stimuli	Electrocutaneous	8 (36.36)
	Thermal	9 (40.91)
	Pressure	3 (13.64)
	Other†	2 (9.09)

* One study^38^ used both direct and indirect methods of presenting the model.

† Two studies classified as “other”^46,47^ did not use a traditional noxious stimulation; instead, they used an inert sham toxin that simultaneously served as a placebo.

OL, observational learning.

#### 3.2.2. Placebo and nocebo effects in experimental pain models

##### 3.2.2.1. Effects of observational learning on pain

Nineteen articles (including one that described 2 individual experiments) investigated the effects of OL on noxious stimulation in experimental pain models. The most common modality of noxious stimulation was thermal (either hot or cold; 40.91%, k = 9), closely followed by electrocutaneous stimulation (36.36%, k = 8) and pressure pain (22.73%, k = 3). Full details on the studies that were included in the systematic review are available in Table 2. For the thermal stimulation, heat application to the skin (k = 5), cold bars (k = 1), the cold pressor task (k = 1), or the warm water task (k = 1) was used. In 10 studies (52.63%), the authors indicated that they investigated placebo effects; of these, 9 (90.00%) reported significant placebo effects elicited by OL. Six studies (31.58%; 7 experiments) investigated nocebo effects; of these, 3 (50.00%) reported that OL induced significant nocebo effects.

**Table 2:** Characteristics of studies included in the systematic review.

Author and year	City, county	# Evaluated participants	# Males	Age at enrolment: Mean (SD)	Trial design	Was there any form of baseline assessment/calibration?	Type of effect aimed to be induced by OL (as specified by authors)	The way the pain-related information is presented to the participant		Sex of the model	Type of a placebo	Type of placebo	Nature of noxious stimuli	Study included in the meta-analysis
Bajcar et al., 2020^7^	Kraków, PL	96	36	22.00 (2.67)	Parallel groups	No/yes	Placebo	In-person	Direct	F	Color	Nonmedical	Electrocutaneous	Yes
Bajcar et al., 2022^6^	Kraków, PL	150	99	23.06 (3.43)	Parallel groups	Yes/yes	Placebo	Pain ratings from others	Indirect	Both	White circle	Nonmedical	Thermal (heat)	Yes*
Bieniek and Bąbel, 2022^14^	Kraków, PL	60	28	23.38 (2.55)	Parallel groups	No/yes	Placebo	Videotaped	Indirect	M	Color	Nonmedical	Electrocutaneous	Yes
Brączyk and Bąbel, 2021^17^	Kraków, PL	60	24	23.65 (2.54)	Parallel groups	No/yes	Placebo	Videotaped	Indirect	M	Color	Nonmedical	Electrocutaneous	Yes
Colloca and Benedetti, 2009^21^	Turin, IT	16	0	21.70 (3.40)	Parallel groups	No/yes	Placebo	In-person	Direct	M	Color. Sham electrode	Medical	Electrocutaneous	Yes
Egorova et al., 2015^27^	Boston MA	20	8	23.00 (2.00)	Within-subjects	No/yes	Both	Videotaped	Indirect	Both	Shapes	Nonmedical	Thermal (heat)	Yes†
Helsen et al., 2011^33^	Leuven, BE	62	0	19.80 (1.80)	Cross-over	No/NA	Nocebo	Videotaped	Indirect	F	Color	Medical	Thermal (CPT)	Yes
Helsen et al., 2013^34^	Leuven, BE	60	0	19.88 (2.68)	Within-subjects	No/NA	Nocebo	Videotaped	Indirect	F	Colored water	Nonmedical	Thermal (WWT)	Yes
Helsen et al., 2015; exp 1^35^	Leuven, BE	49	0	20.47 (3.76)	Within-subjects	No/NA	Nocebo	Videotaped	Indirect	F	Color	Nonmedical	Thermal (cold bars)	Yes
Helsen et al., 2015; exp 2^35^	Leuven, BE	43	0	20.16 (1.65)	Within-subjects	No/NA	Nocebo	Videotaped	Indirect	F	Color	Nonmedical	Thermal (cold bars)	Yes
Hunter et al., 2014^38^	London, UK	45	0	28.37 (7.46)	Parallel groups	No/yes	Placebo	Both live and videotaped	Both	F	Color. Sham electrode	Medical	Electrocutaneous	Yes
Raghuraman et al., 2019^56^	Baltimore MD	31	12	23.40 (4.00)	Within-subjects	No/yes	Placebo	Pictures	Indirect	M	Color. Colored cream	Medical	Thermal (heat)	Yes
Schenk and Colloca, 2020^60^	Baltimore MD	38	15	28.10 (8.50)	Within-subjects	No/yes	Placebo	Videotaped	Indirect	M	Color. Colored cream	Medical	Thermal (heat)	Yes
Świder and Bąbel, 2013^65^	Kraków, PL	84	42	22.70 (2.69)	Parallel groups	No/yes	Placebo	In-person	Direct	Both	Color	Nonmedical	Electrocutaneous	Yes
Świder and Bąbel, 2016^66^	Kraków, PL	65	0	22.23 (2.64)	Parallel groups	No/yes	Placebo	In-person	Direct	F	Color. Geometric shape	Nonmedical	Electrocutaneous	Yes
Vögtle et al., 2013^69^	Göttingen, DE	53	0	22.50 (4.40)	Parallel groups	No/NA	Nocebo	Videotaped	Indirect	F	Cream	Medical	Pressure	Yes
Vögtle et al., 2016^70^	Göttingen, DE	97	0	43.05 (15.51)	Parallel groups	No/NA	Nocebo	Videotaped	Indirect	F	White cream	Medical	Pressure	Yes
Vögtle et al., 2019^71^	Göttingen, DE	80	0	22.40 (4.80)	Parallel groups	No/NA	Nocebo	Videotaped	Indirect	F	White cream	Medical	Pressure	Yes
Lorber et al., 2007^46^	Mansfield CT	86	35	18.99 (1.02)	Parallel groups	Yes/NA	Nocebo	In-person	Direct	F	Sham toxin	Medical	n/a	No‡
Mazzoni et al., 2010^47^	Hull, UK	119	60	20.67 (4.63)	Parallel groups	No/NA	Nocebo	In-person	Direct	Both	Sham toxin	Medical	n/a	No‡
Tu et al., 2019^67^	Boston MA	21	9	25.00 (3.90)	Within-subjects	No/yes	Both	Videotaped	Indirect	Both	Neutral male faces	Nonmedical	Thermal (heat)	No§
Zhang et al., 2017^73^	Chongqing, China	40	0	20.57 (1.46)	Parallel groups	No/yes	Both	Videotaped	Indirect	M	Red/green dot. Metal ring	Nonmedical	Electrocutaneous	No‖

* Pain ratings from others were presented in 2 ways across groups: without any observation of the model (ratings only) and with indirect observation of the model (ie, on a picture). Only the subgroups in which pain ratings were combined with a picture were included in the meta-analysis in the subgroup “indirect observation of the model.”

† Both placebo and nocebo were induced; study excluded from certain subgroup analyses.

‡ No pain stimulation comparable to other studies; headache assessed as a result of placebo manipulation.

§ Observation was reinforced by a conditioning procedure.

‖ Participants also underwent a conditioning procedure that was intermixed with OL so the pure OL effect could not be isolated.

CPT, cold pressor task; F, females; M, males; NA, not applicable; OL, observational learning; WWT, warm water task.

Three studies reported that they investigated both placebo and nocebo effects evoked by OL. One study reported significant placebo and nocebo effects when comparing pain that was experienced following the presentation of a low-pain–associated or high-pain–associated cue with pain after a neutral cue was presented. The effect sizes of the placebo and nocebo effects were not reported. Two other studies, although they did report significant placebo and nocebo effects, mixed observational learning with other learning strategies (eg, conditioning), which complicates identifying the effect of OL alone. Of note, the findings of these 2 studies with mixed approaches seem to suggest that significantly larger nocebo effects than placebo effects were induced.

##### 3.2.2.2. Effects of observational learning on expectancy

Seven of the 19 articles (36.84%; including 8 experiments) investigated the impact of OL on self-reported expectations. The majority of them (k = 6; 75.00%) reported that OL influences expectations. One article did not report any statistical test for the effect of OL on pain expectation; however, it did note that expectancy was significantly associated with the magnitude of OL-induced changes in pain. Finally, a study on nocebo OL reported no associations between expectancy and pain intensity.

Interesting and potentially relevant for the role that expectancy may play in OL-evoked placebo and nocebo effects are the 2 studies that have investigated subliminal evocation of placebo and nocebo effects after OL. One reported that placebo but not nocebo effects could be evoked subliminally. This would suggest that expectations do not necessarily need to be explicit and verbalized but that placebo effects after OL at least may also be evoked by nondeclarative processes. Conversely, the other study demonstrated that neither significant placebo nor nocebo effects could be evoked subliminally.

##### 3.2.2.3. Effects of observational learning on other outcome parameters

Several other outcomes were assessed in the studies. Briefly, OL induced changes in pain-related fear or anxiety in all studies that assessed this outcome after OL (k = 5; 100.00%). Two of 4 studies (50.00%) reported that OL elicited changes in experienced pain unpleasantness. Regarding physiological parameters, 1 of 3 studies (33.33%) reported differences in skin conductance responses to exposure to low-pain–associated and high-pain–associated cues. Another study assessing heart rate demonstrated heart rate acceleration for hypoalgesia-associated cues and deceleration for nonhypoalgesia-associated cues.

Three of 19 studies (15.79%) investigated the neural pathways and brain areas involved in observationally induced effects on pain. One of these studies employed electroencephalography (EEG), another resting-state magnetoencephalography (MEG), and the last a functional magnetic resonance imaging (fMRI) paradigm. In short, smaller event-related potentials in locations that are associated with frontal attentional processes,^56^ decreases in alpha band frequency between the left rostral anterior cingulate cortex (rACC) and left middle temporal gyrus (MTG) that reflect top–down control processes and a role of information processing,^67^ and higher connectivity between the dorsolateral prefrontal cortex (DLPFC) and temporoparietal junction (TPJ) were associated with OL-induced placebo effects.^60^

##### 3.2.2.4. The role of empathy in observational learning–evoked placebo and nocebo effects

In 8 of 19 papers (42.11%; 9 experiments) associations between OL-induced differences in pain and trait empathy were investigated. Four studies report no significant associations between trait empathy and OL-induced effects in pain at all. Three report that higher empathic concern is associated with a larger magnitude of the (video-based) OL-induced effects, and one other study found this association only for in-person observation. Higher personal distress was found to play a role in observational learning in 2 studies (although only marginally in one of them), and a single study reported a role of higher perspective taking.

#### 3.2.3. Placebo and nocebo effects in pain induced in other models

Two studies investigated the effects of observational learning on pain outside of experimental (topical) pain induction paradigms. Within these studies, a model of psychogenic illness was employed. Participants observed a model inhaling a sham environmental toxin, and modelling side effects from these toxins, including headache. When participants inhaled the sham toxin themselves, those who had observed the symptoms in the model were more likely to report them.

#### 3.2.4. Methodological aspects of observational learning

The placebo or nocebo effect induction itself was typically done by having the observer watch a model (ie, a demonstrator) who rated pain as either high or low, following, for instance, administration of a placebo cream or presentation of an abstract cue. The model was predominantly presented through a video recording or pictures (ie, symbolic modelling; 68.18%, k = 15). Six studies presented the model in-person (ie, behavioural modelling; 27.27%). One study (4.55%) presented the pain ratings of the model on the screen as follows: in some groups, the pain ratings were presented without observation of the model (ie, verbal modelling); in other groups, this was combined with observation of the model (ie, on a picture). Half of the studies included only a female model (k = 11).

Approximately half of the studies used medically connoted placebos (45.45%, k = 10), and others used abstract cues (54.55%, k = 12). Color cues presented on a monitor (31.81%, k = 7) and cream application (13.64%, k = 3) were most commonly used. The other types of placebos included neutral male faces (4.55%, k = 1), red/green dots paired with a metal ring (4.55%, k = 1), colored water (4.55%, k = 1), geometric shapes (4.55%, k = 1), inert sham toxin (9.09%, k = 2), sham electrode (9.09%, k = 2), or combinations of some of the above.

### 3.3. Risk of bias

The included studies mostly had low (k = 6) or moderate (k = 14) bias scores (Supplementary Table S4, available at http://links.lww.com/PAIN/B845). The moderate-bias studies mainly owed their lower ratings to a lack of information on whether sample participants represented the wider population from which they were recruited, whether the study was single or double blinded, or if the study had sufficient power to detect a clinically important effect. One study^27^ was rated as having high potential bias, mainly due to the fact that few details were available concerning the blinding of the study participants, their characteristics, and the distribution of the principal confounders.

### 3.4. Meta-analysis

For the meta-analysis, only those studies that investigated the changes induced by observational learning exclusively in experimental pain models were included.

#### 3.4.1. Magnitude of the effects induced by observational learning for pain

**Figure 2.**
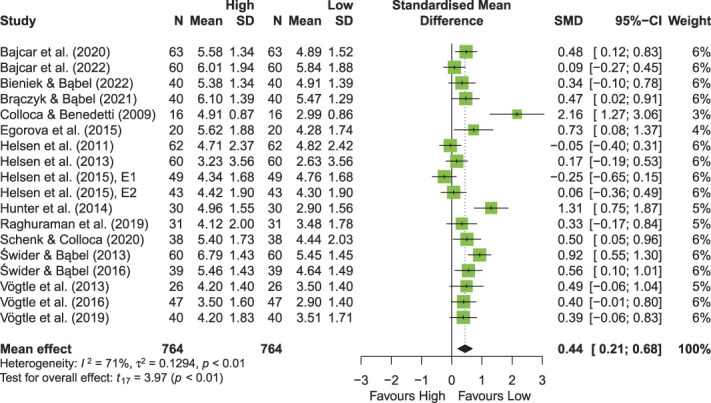
Forest plot of the random-effects model comparison of the magnitude of placebo and nocebo effects, observed across the 19 experiments included in the meta-analysis. 95% CI, 95% confidence interval; SMD, standardized mean difference.

Observational learning was generally effective in modulating pain experience (Fig. 2). The mean effect of OL on placebo and nocebo effect magnitudes in pain, ie, the standardized mean difference in pain ratings between high-pain and low-pain cues, was 0.44 pooled across the studies (95% CI 0.21; 0.68, *P* < 0.01). The funnel plot with effect sizes for pain showed a slightly skewed distribution, and the Egger's test was statistically significant (t [16] = 3.33, *P* = 0.0042, intercept = −0.8753), which indicates possible publication bias (Supplementary Figure S1, available at http://links.lww.com/PAIN/B844).

A subgroup analysis showed that the effect of OL was significantly larger for studies that the authors labelled as placebo in comparison to studies labelled as nocebo studies (*P* = 0.02; Supplementary Figure S2, available at http://links.lww.com/PAIN/B844). The magnitude of the OL-induced placebo effect was significant across studies (0.64, 95% CI 0.26; 1.02, *P* < 0.01). However, the mean effect of the OL-induced nocebo effect was not significant (0.15, 95% CI −0.10; 1.02, *P* = 0.19). Tentatively, these findings seem to suggest that placebo effects, but not nocebo effects, can be elicited with OL (see Supplementary Appendix 1, which contains further subgroup analyses of placebo vs nocebo, available at http://links.lww.com/PAIN/B844).

#### 3.4.2. Effect of the way the model is presented on the magnitude of the observationally induced effects

Observational learning had a significantly larger effect on pain ratings when the model was shown in-person instead of on video (*P* < 0.01), with one study using a combined mode of observation. In the subgroup where the model was presented to the participants in-person, the pooled mean effect of OL-induced effect magnitude was 0.94 but was not significant (95% CI −0.19; 2.08, *P* = 0.08), whereas the pooled SMD was significant but lower by 0.73 points (0.21 [95% CI 0.06; 0.37], *P* = 0.01) when the model was presented through photographs or a video recording. A visual representation is shown in Supplementary Figure S3, available at http://links.lww.com/PAIN/B844.

#### 3.4.3. Effect of placebo type on the magnitude of the observationally induced effects for pain

No statistically significant differences in placebo or nocebo effect magnitude were found between placebo types (*P* = 0.23; Supplementary Figure S4, available at http://links.lww.com/PAIN/B845): the magnitude of effects was generally similar, regardless of whether the placebo had medical (eg, a cream; SMD = 0.62 95% CI 0.08; 0.58, *P* < 0.03) or abstract connotations (ie, nonmedical, eg, colors or geometric shapes displayed on a computer monitor; SMD = 0.31 95% CI 0.05; 0.58, *P* < 0.03).

#### 3.4.4. Magnitude of the effects induced by observational learning for expectancy

**Figure 3.**
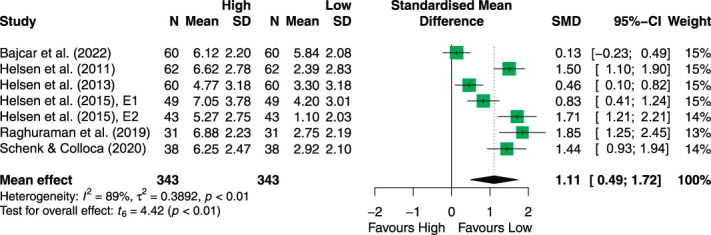
Forest plot of the random-effects model comparison of the effect of OL-induced effects on expectancy ratings. Of all 19 studies on observational learning, 7 investigated the impact of OL on expectancy. 95% CI, 95% confidence interval; OL, observational learning; SMD, standardized mean difference.

Observational learning was shown to effectively modulate pain expectancy ratings. The mean overall effect of OL on expectancy ratings (ie, the SMD between high and low cue expectancy ratings) was 1.11 pooled across the studies (95% CI 0.49; 2.04, *P* < 0.01). These results are summarized in Figure 3.

#### 3.4.5. Associations between empathy and observationally induced placebo effects

The empathic concern subscale of the Interpersonal Reactivity Index was significantly positively correlated with the magnitude of observationally induced placebo and nocebo effects (random-effects model correlation coefficient = 0.14 [95% CI 0.01; 0.27], *P* = 0.03). These findings suggest that OL was more effective when empathic concern was higher. No significant correlations with the other 3 subscales of the IRI were found (all *P* ≥ 0.068; Table 3).

**Table 3:** Correlation between observationally induced placebo and nocebo effects and empathy ratings.

IRI subscale	Correlation coefficient	95% CI	*P*
EC	0.14	0.01; 0.27	0.0341
PT	0.04	−0.11; 0.18	0.5684
PD	0.08	−0.07; 0.21	0.2398
F	0.08	−0.01; 0.17	0.0680

Empathy results are based meta-correlations calculated for 8 studies: Bajcar et al., 2020,^7^ Colloca and Benedetti, 2009,^21^ Raghuraman et al., 2019,^56^ Świder and Bąbel, 2013^65^ and 2016,^66^ Vögtle et al., 2013,^69^ 2016,^70^ and 2019.^71^

95% CI, 95% confidence interval; EC, empathic concern; F, fantasy; IRI, interpersonal reactivity index; PD, personal distress; PT, perspective taking.

### 3.5. Sensitivity analysis

We performed a sensitivity analysis by excluding one study^38^ from the assessment of the magnitude of placebo effect induced by OL. Its experimental manipulation featured additional instructions that could have amplified the effects of OL, as they mentioned the explicit analgesic effect following green cues. Although these instructions were not sufficient grounds for exclusion from the meta-analysis (ie, there are many instances in which researchers call attention to potential contingencies between cues and effects on pain, and not all researchers publish the full instructions they provided to participants), we felt that it was worth controlling for the impact of this particular study on our analysis given that the instructions were suggestive in nature. A nonsubstantial change in the mean effect of OL across all the analyzed studies was observed; however, the overall effect of the placebo/nocebo manipulation was 0.39 (95% CI 0.17; 0.61) after exclusion of 2 aforementioned studies vs 0.44 (95% CI 0.21; 0.68) without exclusion. The observed effect remained at the same level of significance as before the exclusion (*P* < 0.01).

## 4. Discussion

The current systematic review and meta-analysis aimed to summarize the findings of studies on the efficacy of observational learning in evoking placebo hypoalgesia or nocebo hyperalgesia. Overall, observational learning was found effective in modulating pain experience and self-reported pain expectancy. A subgroup analysis of studies purported by the authors as inducing placebo effects vs those inducing nocebo effects demonstrates that placebo effects but not nocebo effects can be induced by OL. However, the majority of the included studies did not use a within-subject condition as a comparator for post-OL decreases or increases in pain in the experimental conditions (for instance, a no-observation within-subject control condition for pain or baseline assessment of pain). Because of this, the meta-analytical between-study comparison of the magnitude of placebo vs nocebo effects may not be reliable and should be treated with great caution. The type of observation (ie, in-person vs videotaped) modulated the magnitude of OL effects on pain. No differences in pain modulation were detected between studies that used medically connoted placebos or nonmedical placebos. Finally, of all the empathy-associated factors, only the empathic concern subscale was significantly associated with OL effect magnitude, with greater pain modulation occurring when observers' empathic concern was higher.

That pain experience following an inert intervention can be altered by OL is in line with our hypothesis and with other lines of research that show that somatic symptoms can be modulated by this type of learning.^19,46^ Overall, the meta-analysis demonstrates a medium-sized effect of OL on pain. However, the magnitude of this effect varies across studies: with some studies reporting large effects^21,27,38,65^ and other studies reporting no to small effects.^6,33–35,56^ Methodological differences between the studies could explain some of the variance in OL effect magnitude. For instance, some of these differences could be found in choices relating to the content and form of OL, whereas others relate to the pain model. To illustrate, some studies investigated OL that included the observation of verbally rated pain,^7,14,17,21,60,65,66,69,70^ whereas other studies exclusively used the model's facial expression of pain as a cue for the effectiveness of the placebo in observational learning.^33–35,71^ Although these differences in OL content were not meta-analyzed in particular, the qualitative comparison shows that studies that use verbal ratings generally appear to obtain effects of a bigger magnitude relative to those studies that use pain expression (see Table 2, Supplementary Table S6, available at http://links.lww.com/PAIN/B845). Given that facial expression of pain is a nonverbal cue, it may be more open to interpretation.^54^ Not every individual may be equally adept at picking up on more or less subtle nonverbal pain cues in such an experimental setting. Moreover, there is evidence that observers demonstrate a systematic underestimation bias when evaluating pain expressions.^40,55,57^ Finally, although it was indicated in some studies^27,56^ that verbal ratings were combined with facial pain expression, it is not clear to what extent the models in the studies on OL with verbally expressed pain also displayed facial pain expressions.

Other methodological differences between studies include the experimental pain model that was used. Although all of the models have been previously validated in healthy volunteers, they do differ between OL studies. For instance, some use short-term topical noxious stimuli, such as heat^6,27,56,60^ or cold^35^ stimuli, electrical (ie, electrocutaneous) stimuli,^7,14,17,21,38,65,66^ or mechanical pressure.^69–71^ Finally, some studies used cold pressor tests^33^ or warm water immersion tests.^34^ The involved tissues and underlying neurobiological pathways that generate the pain sensations may differ between these experimental pain models.^50,63^ Given that some biological pathways (eg, antinociceptive activation of µ-opioid receptors in the periphery^12^) may be specifically involved in placebo hypoalgesia, differences in pain modalities could influence the efficacy of OL in eliciting placebo or nocebo effects in pain.

No clear distinction between placebo and nocebo effects generated by observational learning could be made based on the included studies' methodology because most studies that were included compared the effects of cues (ie, cream, color) associated with high pain relative to cues associated with low pain (see Supplementary Table S6, available at http://links.lww.com/PAIN/B845). Instead, the current meta-analysis attempted to systematically compare OL between studies in which, according to the authors, the aim was to induce placebo hypoalgesia and those whose aim was to induce nocebo hyperalgesia. The subgroup analysis shows that the magnitude of the effect of OL on pain is significant only in the studies that are purported to elicit placebo effects. The nocebo studies demonstrate nonsignificant effects of a smaller magnitude. However, any conclusions derived from these results need to be seen as tentative. It is unclear how much information these analyses can actually provide about the exact magnitude of placebo effects and nocebo effects induced by OL, given the similarity of the methodology of the studies included in either the placebo or nocebo subgroups. For example, in most of the (placebo) studies, participants observed the model providing high and low pain ratings for 2 distinctive cues, respectively.^6,7,14,17,21,38,56,60,65,66^ In other studies that were most often defined as nocebo studies, participants observed a model's facial expression of pain, or lack thereof, combined with either 2 distinct cues^33–35^ or a single placebo cue.^69–71^ Even in the event of the latter, one may argue that a model with a neutral expression in the absence of a placebo cue could be interpreted as not being in pain. Thus, both observations contain information about the nature of the noxious stimuli.

Although these designs can, without question, demonstrate the efficacy of OL in modulating pain in general, they do so by comparing a low relative to a high pain condition, which complicates any estimation of whether the pain modulation is ultimately a nocebo effect or a placebo effect. It is not clear how much pain participants would have experienced in the absence of the observational learning because the included studies lacked either a baseline assessment or a comparison with pain experience following a neutral nonpaired cue. So far, only a single study has included a baseline assessment for comparison,^6^ and one other study involved a comparison with a neutral cue.^27^ Although the latter would provide a good estimate of whether magnitudes of OL-induced placebo and nocebo effects differ, no effect sizes were reported for these comparisons. Moreover, in another study,^65^ it was demonstrated that, in comparison with a no-observation control condition, pain only increased for cues that were previously paired with a high pain rating given by the model, whereas pain remained stable over time for the cues paired with low pain ratings given by the model. This could indicate that what authors may see as placebo effects could, in fact, be masked nocebo effects. Future research is needed to investigate these effects more closely and to compare the magnitude of placebo and nocebo effects elicited by OL not only amongst themselves but also with a baseline assessment of pain or a neutral control condition. Using an adequate control for natural changes in pain over time may shed more light on the direction of the effects that OL induces in pain. Unravelling the direction of these effects is important, given multiple reasons. For one, placebo and nocebo effects have different implications for clinical practice (eg, we may want to enhance placebo effects but prevent nocebo effects^28^). Moreover, although placebo and nocebo effects share many psychological mechanisms, such as learning and expectations, they are in fact distinct effects that involve different brain regions.^29,30^ Comparing placebo and nocebo effects induced through OL, and in particular identifying the ways in which these effects may differ, therefore remains a priority.

Significant differences in the magnitude of OL-induced effects were found for the type of observation (in-person vs videotaped). Notably, in-person observation of a model resulted in large but ultimately nonsignificant effects of OL on pain. Conversely, observation of a videotaped model led to a smaller but significant effect. Prior frameworks indicated that the manner in which previous models were videotaped could have limited the amount of information available to the observer.^5^ This may mean that the effects elicited through observation by video could be smaller than those elicited through in-person observation. Although this holds true in our meta-analysis, at least when looking at the absolute value of the SMD, a larger between-study variance was detected in those studies that used in-person models. Some of the studies^21,65^ show large effects of in-person OL of a model, whereas other studies show moderate effects.^7,66^ Ultimately, the effects of OL on pain in this subgroup were nonsignificant. This seems to suggest that when it works, in-person observation of a model could potentially be more powerful than observation of a model presented through video; however, this also seems to suggest that it may be more difficult to achieve such an effect. It should be noted though that there are fewer studies that use in-person models than those that use videotaped models, and these findings may change when more data are available. An experimental study that compared the 2 types of modelling reported finding effects of a similar magnitude on pain.^38^ Future studies may also take a closer look to how the models are introduced. When the instructions call attention to the contingency between the cues and pain stimuli, this could enhance OL efficacy. In-person observation may also increase the relevance to the participant, whereas video-based observation could result in smaller effects when the relevance and contingency between cues and stimuli are not explained. To some extent, these effects may depend on the type of model that is used. Some studies reported using a male model,^14,17,21,56,60^ whereas other studies used a female model (including but not limited to Refs. 7, 38, 69, 70, 71). As demonstrated by Świder and Bąbel,^65^ the sex of the model can impact the magnitude of the effects elicited by observational learning, with larger effects being found for male models. Potentially, other as-yet uninvestigated differences between models could influence the efficacy of OL, for instance, differences in personality traits or social status. To date, only 3 other studies^7,14,17^ have investigated the effects of a model's traits on the magnitude of OL. Other differences between models that may influence OL efficacy remain largely unidentified. So far, these studies indicate that effects can be induced by OL regardless of the model's traits, but these traits do seem to moderate the magnitude of the effect. In addition, sex and racial concordance may contribute to placebo and nocebo responding in observational learning. Although it was not possible to investigate the influence of sex concordance with a meta-analytical approach given that the male-to-female ratio of the participant groups and the sex of the model were skewed towards females, this needs to be investigated systematically in the future. Moreover, females may be more sensitive to OL-induced placebo effects than males.^16,65^ It is recommended from both a methodological and diversity perspective that participant groups for pain and placebo studies are diverse, also with regard to sex and gender.^15,51^ Finally, only one study so far has investigated whether verbal modelling (ie, observing other people's pain ratings) can trigger placebo and nocebo effects. Although there is evidence that this type of (social) information can affect pain experience directly,^42,43,52^ these studies did not involve a placebo or nocebo effect induction (ie, with a sham intervention) but rather investigated the direct impact on pain. Studying how written pain information influences pain in the context of placebo effects is relevant, given the amount of information on other people's experiences with pain treatment that is available, for instance, on social media and the internet.

The type of placebo did not modulate the magnitude of the observationally learned effects in pain. Both studies that used medically connoted placebos and those that used abstract cues found that OL significantly modulates pain experience. This finding is not wholly unexpected, given that placebo and nocebo effects can be elicited by the entire psychosocial context or by parts of this context.^48,58^ That the placebo types do not influence the magnitude of OL supports this broad understanding of placebo effects as elicited by any contextual cue. It should be noted that of those studies that used medically connoted placebos, almost all of them included a dual manipulation (ie, 2 [colored] creams were used). Only 4 studies so far have used a single placebo, of which one studied placebo hypoalgesia elicited by an abstract cue,^6^ and 3 aimed elicit nocebo hyperalgesia through a cream and compare it with a no-cue control condition.^69–71^ Not all of the latter studies yielded a significant effect of OL. Although this may be partially because of the type of manipulation (eg, Vögtle et al.^71^ used facial pain expression, which, as previously discussed, may be less effective), it could also mean that placebo effects may be relatively more easy to associate with medically connoted placebos than nocebo effects. Future research should systematically compare the two because the methodologies vary too much across these studies to reliably draw conclusions at this time.

Expectancy was modulated by OL. Although on the group level, these effects appear to be in the same direction (ie, more pain is expected for the high-pain cues relative to the low-pain cues), we were unable to associate them with the incidence of pain modulation through OL because most studies did not report correlational data for expectancy and pain ratings. Only one study reported a positive association between the effects of OL on expectancy and its effects on pain.^6^ These results indicate that expectancy is likely related to the magnitude of placebo effects elicited by OL. Another study reported no associations between expectancy and pain intensity following nocebo OL.^71^ Because OL was not effective in modulating pain in this study, the association between expectancy and nocebo hyperalgesia may be underestimated. Investigating how changes in expectancy are associated with changes in pain experience is important for future work. Speculatively, those people who expect a pain reduction or increase following OL are not necessarily those who may experience it and vice versa. For instance, following a classical conditioning procedure, nocebo effects were induced in the absence of conscious expectancies.^4^ In another study, prior therapeutic experience but not expectancy was predictive of placebo effects.^20^ These findings contradict models where expectancy fully mediates conditioned placebo effects. Such a notion may also be relevant for placebo effects elicited by other types of learning, including OL. Moreover, it could speculatively be possible that, depending on the specific content of the placebo or nocebo modulation, some methods could result in expectancy violations.^37^ It has been shown that expectancy violations can reduce placebo and nocebo effects when they are shaped by classical conditioning.^23^ As of now, it is unclear whether expectancy violations can occur in OL-induced placebo and nocebo effects. The type of placebo may be relevant here because creams, ointments, or other medically connoted placebos could trigger expectancies that may not be in line with experimental nocebo manipulations.

Empathy was only partially associated with the magnitude of observationally learned effects in pain, as evidenced by a small but significant association between empathic concern and pain modulation, and the lack of an association between the other empathy subscales and the effects of OL on pain. Relatively few studies have assessed participants' empathy and associated this trait with experienced placebo hypoalgesia or nocebo hyperalgesia following OL, though, and future studies should aim to broaden the evidence base for the role of empathy in observationally learned placebo and nocebo effects. Moreover, given that the studies included relatively homogeneous participant samples, it may be possible that there was too little variance in empathy to assess its impact on OL efficacy. Empathy may also be tied to a specific person or situation. This situational empathy, or state empathy, may be of interest for future OL studies.

Finally, although the current meta-analysis aimed to include patient studies, the search strategy failed to yield studies conducted in this population. Instead, almost all of the studies used a relatively young and healthy participant sample. Studying whether and how observational learning or, more broadly speaking, social information could influence chronic pain and the efficacy of pain treatment is important given the influence that placebo and nocebo effects may have in pain-related medical conditions. Considering the large impact of chronic pain on individuals' well-being and society as a whole, endeavoring to do so may be particularly relevant. Future studies should investigate the impact of OL on the pain that patients experience, thereby validating the OL model in this population. A recently published study showed that observational learning (ie, observing beneficial treatment outcomes in another patient) does not reduce chronic low back pain but that it does reduce perceived disability in a sample of patients.^62^ New knowledge generated by research in patient populations could then help to improve existing treatments and pain management in chronic medical conditions. In particular, learning how negative social information can impact the efficacy of and adherence to treatment for chronic pain could be of help to develop novel strategies that deal with this type of information. To enhance placebo hypoalgesia, information that patients receive before starting a new treatment could, for instance, include individual accounts of prior treatment successes. Moreover, it is advised that healthcare professionals take into account the impact that observations of successful treatment in others may have on their patients.^11^ This is particularly relevant in current times, considering the large influence and reach of social media platforms and how common online searches for health-related information are.^64,72^

To our knowledge, this is the first systematic review and meta-analysis of placebo hypoalgesia and nocebo hyperalgesia elicited through observational learning. The findings of the current meta-analysis provide further support for the model of observational learning as a mechanism of placebo and nocebo effects that was proposed by Bajcar and Bąbel.^5^ Moreover, it identifies existing knowledge gaps in the literature. Several limitations of the meta-analysis need to be acknowledged. First, some of our research questions could not be answered fully or only very tentatively at best. For instance, no data on associations between expectancy and pain modulation by OL were available. Some study end points fell outside the scope for comparison on a meta-analytical level. These include subjective ratings (such as fear and pain unpleasantness), physiological parameters (skin conductance, heart rate), and brain imaging data. Around one-third of the included studies lacked a parallel control group (see Table 2, Supplementary Table S6, available at http://links.lww.com/PAIN/B845). Therefore, the current meta-analysis focused on within-subject comparisons between high-pain and low-pain OL-associated cues. Moreover, when the studies included a parallel no-observation control group, they tended to provide fewer data on the control relative to the experimental groups. For instance, data on expectancy and empathy scores in the control groups were frequently missing.^14,33,35,56,60,69–71^ Finally, the majority of the research presented in this review originates from only 4 research groups, which may affect the OL effects that are reported here. Replication of these findings by other research groups is encouraged.

In short, the current meta-analysis validates the theoretical model proposed previously.^5^ Observational learning is generally an effective way of modulating pain experience and expectancy. The review demonstrates that the manner of observation may influence OL efficacy, as may the observers' empathy levels. Future research should aim to compare placebo and nocebo effects elicited by OL with adequate control conditions to better predict the direction of the effect. Moreover, the OL model needs verification in patient populations. Investigating how OL elicits placebo hypoalgesia and nocebo hyperalgesia and identifying the factors that may influence this process is important for developing novel strategies for dealing with (negative) social information about treatment and pain outcomes.

## Conflict of interest statement

The authors have no conflicts of interest to declare.

## Appendix A. Supplemental digital content

Supplemental digital content associated with this article can be found online at http://links.lww.com/PAIN/B844 and http://links.lww.com/PAIN/B845.

## Supplementary Material
