# Supplementary material for: Learning pain from others: a systematic review and meta-analysis of studies on placebo hypoalgesia and nocebo hyperalgesia induced by observational learning
Source: Pain. 2023 Jun 15;164(11):2383–96. doi: 10.1097/j.pain.0000000000002943 (PMC10578425; doi:10.1097/j.pain.0000000000002943)
Supplement: Supplementary file 1 [file jop-164-2383-s001.pdf]

Supplementary Appendix 1:

Secondary analyses featuring the placebo/nocebo subgroup division

Statistical analysis

Apart from featuring the placebo-nocebo division, the meta-analyses described below are identical to the ones described in the main manuscript: they were based on the random-effect model with the standardised mean difference (SMD) between high-pain-associated cues and low-pain-associated cues for pain intensity (within-groups differences). A positive SMD indicated a bigger magnitude of the effect of OL on either pain or expectancy. The pooled effect was weighted by the sample size in the given study and was estimated with 95% confidence intervals. For empathy, the p-value testing for subgroup differences was based on the random-effect model with between-groups comparison.

RESULTS

Influence of the way the model is presented on the magnitude of the observationally induced effects for placebo and nocebo studies

The effects of OL differed between the subgroup with in-person observation relative to the subgroup with indirect observation ($p < .01$). In the subgroup where the model was presented directly to the participants, the pooled mean effect of OL on the placebo effect magnitude was non-significant (0.94 [95% CI -0.19; 2.08], p=.08), thus showing that direct observation of a model does not result in placebo effects. The pooled SMD was significant but lower by 0.62 points (0.32 [95% CI 0.10; 0.54], p=.02) when the model was presented via photographs or a video recording. We were unable to make a similar comparison for the nocebo effect as the nocebo studies included in our systematic review all presented the model on video. This is shown in Figures A1 and A2.

Figure A1. Forest plot of the random effects model comparison of the effect of the way the model is presented on the magnitude of placebo effects

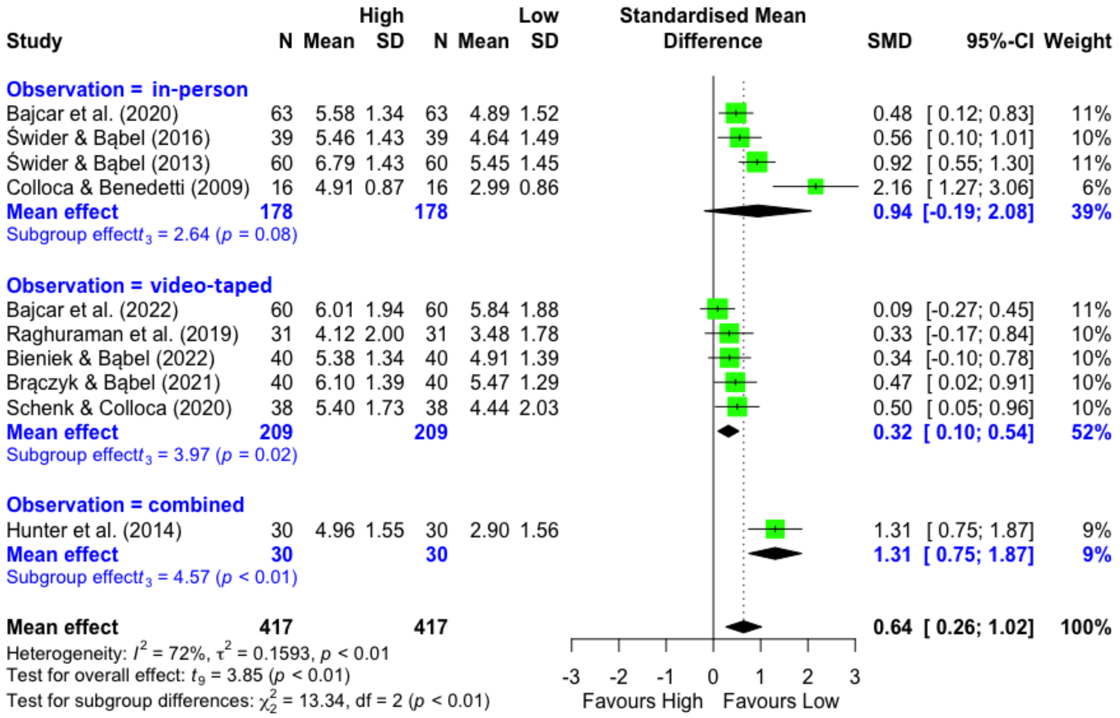

Figure A2. Forest plot of the random effects model comparison of the effect of the way the model is presented on the magnitude of nocebo effects

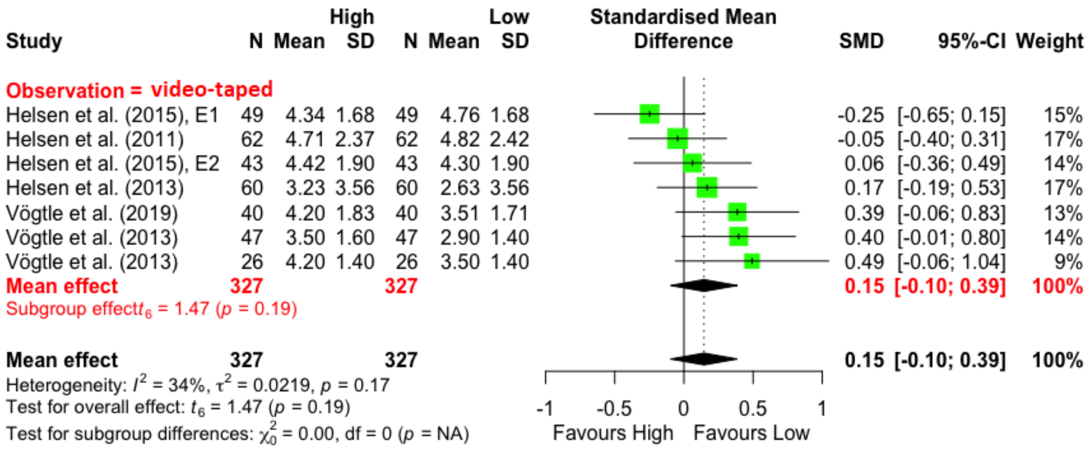

Influence of placebo type on the magnitude of the observationally induced effects in placebo and nocebo studies

The effects of OL in the placebo studies did not differ between the abstract cues and the medically connoted placebos (p=.20). OL effects on pain were significant within the subgroup of placebo studies that used abstract cues ($p < .01$), whereas effects were non-significant for the subgroup with medically connoted placebos (p=.09). This suggests that OL can result in placebo effects when abstract cues are used, but not when medically connoted placebos are used. For nocebo studies, effects were non-significant in both subgroups (both $p > .12$; Figures A3 and A4).

Figure A3. Forest plot of the random effects model comparison of the effect of placebo type on the magnitude of placebo effects

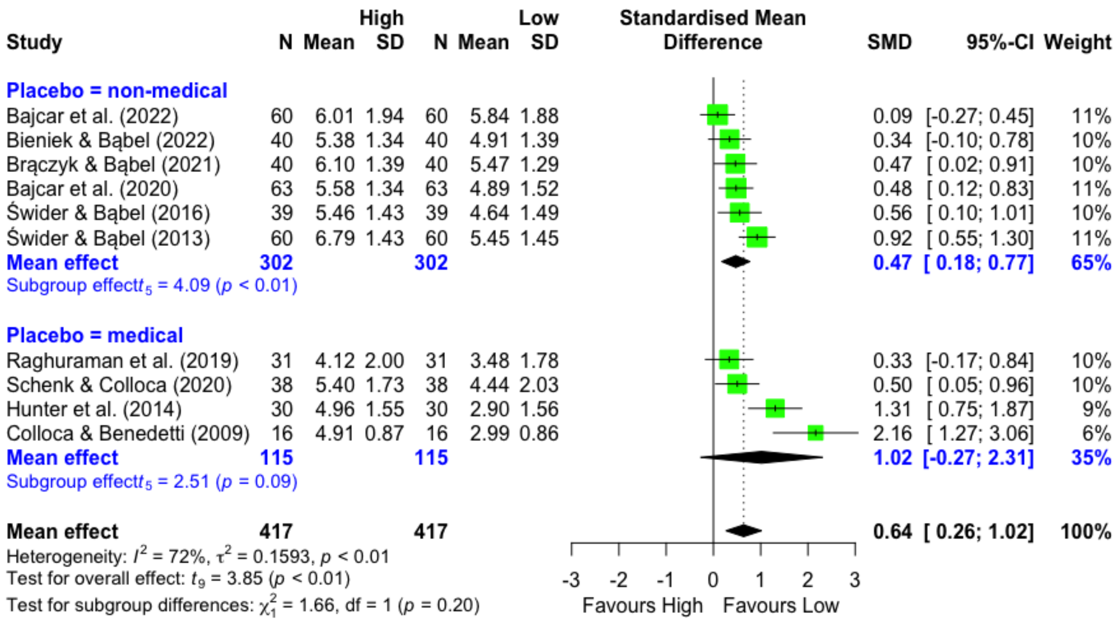

Figure A4. Forest plot of the random effects model comparison of the effect of placebo type on the magnitude of nocebo effects

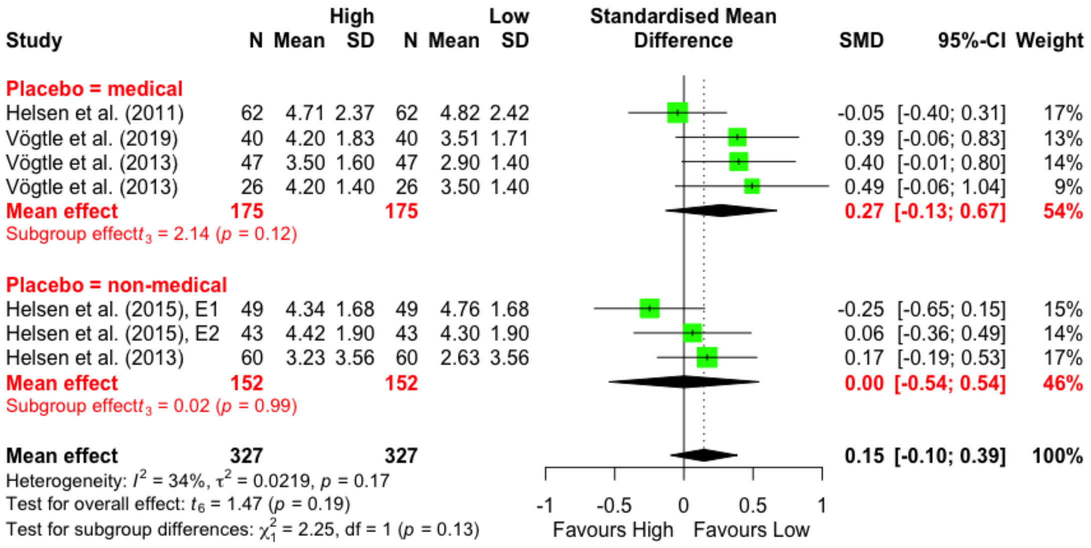

Magnitude of the effects induced by OL for expectancy effects in placebo and nocebo studies

There was no significant difference in the pooled expectancy SMD between the placebo and nocebo subgroups: 1.12 (95% CI -1.12; 3.36) vs 1.11 (95% CI 0.19; 2.04), respectively (p=0.99). However, analysis of the effects of OL on expectancy within each subgroup demonstrates that, overall, OL did not modulate expectancy in the placebo studies (*p*=.17), but it did modulate expectancy in the nocebo studies (*p*=.03). These results are summarized in Figure A5. No associations could be analysed between expectancy and pain as the studies did not report this.

Figure A5. Forest plot of the random effects model comparison of the effect of observationally induced placebo and nocebo on expectancy ratings

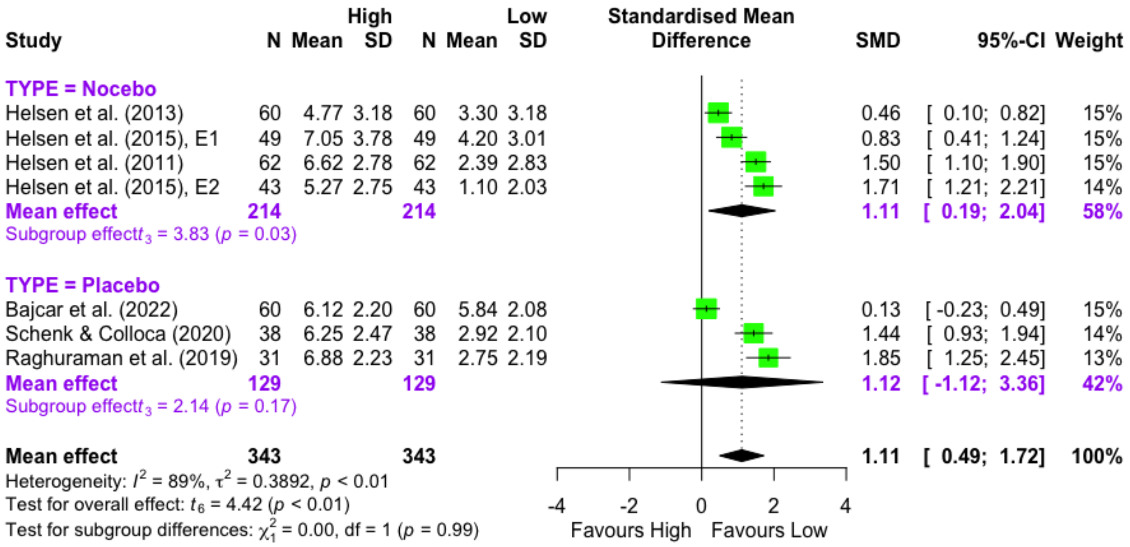

Associations between empathy and observationally induced placebo and nocebo effects

The correlations between the Interpersonal Reactivity Index subscales and the magnitude of observationally induced placebo and nocebo effects did not differ significantly between the studies aimed at inducing placebo effect and the studies aimed at inducing nocebo effect (Table A1). All these correlations were weak (all $r \leq .14$), with the empathic concern subscale of the IRI overall being the most strongly correlated with the observationally induced effect.

Table A1.
Correlation between observationally induced placebo and nocebo effects and empathy ratings.

IRI subscale	correlation coefficient	95% CI	correlation coefficient	95% CI	p
	PLACEBO		NOCEBO		
**EC**	0.14	-0.11; 0.37	0.16	-0.03; 0.35	0.8147
**PT**	0.10	-0.09; 0.28	-0.07	-0.46; 0.34	0.1475
**PD**	0.12	-0.11; 0.34	0.01	-0.39; 0.40	0.3325
**F**	0.03	-0.20; 0.26	0.13	0.01; 0.25	0.1061

IRI – Interpersonal Reactivity Index; EC – Empathic Concern; PT – Perspective Taking; PD – Personal Distress; F – Fantasy

Supplementary Figures

Supplementary Figure S1. Funnel plot. The effect size from each study is plotted against the magnitude of the standard error. The Egger's test was statistically significant (t[16] = 3.33, p = .0042, intercept = -0.8753) indicating for potential asymmetry.

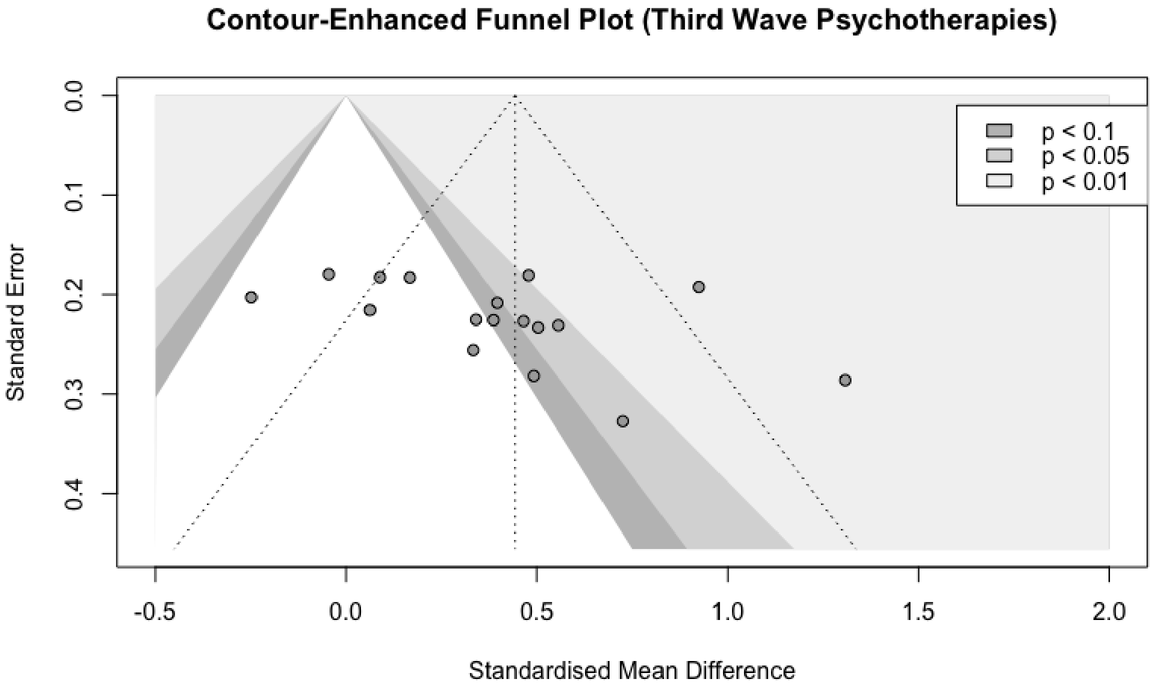

Supplementary Figure S2. Forest plot of the random effects model comparison of the magnitude of placebo and nocebo effects observed across the studies included in the meta-analysis.

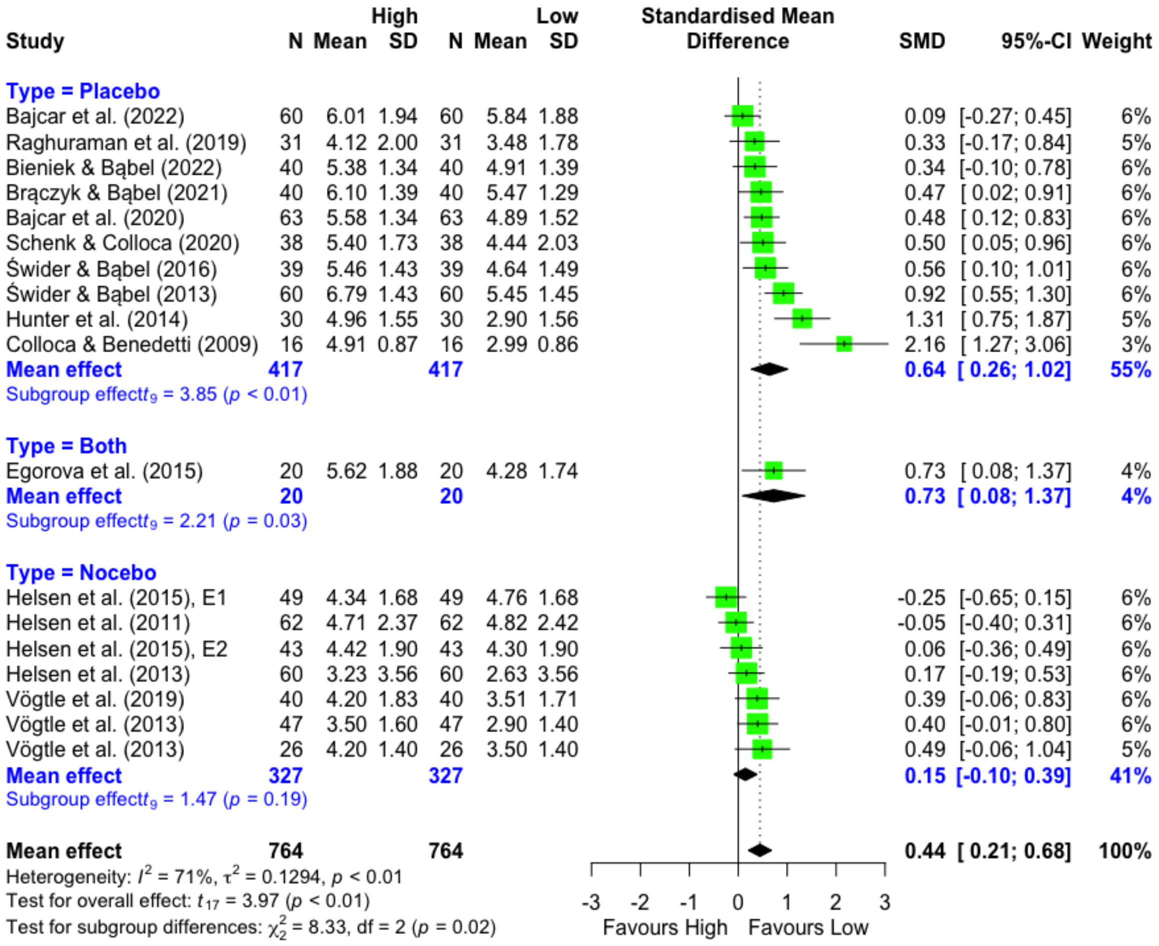

Supplementary Figure S3. Forest plot of the random-effects model comparison of the mode of model presentation on the magnitude of the OBL-induced effect.

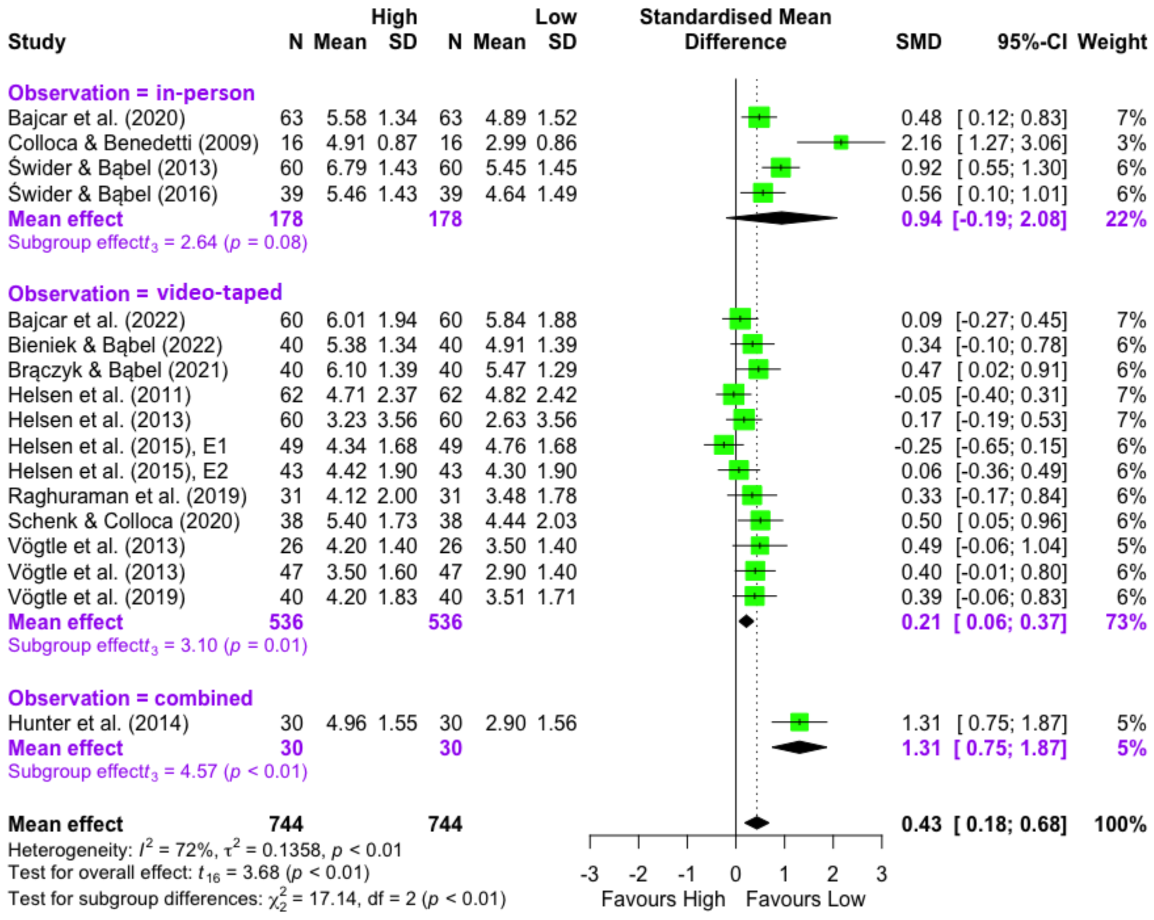

Supplementary Figure S4. Forest plot of the random-effects model comparison of the effect of placebo type on the magnitude of the OBL-induced effect.

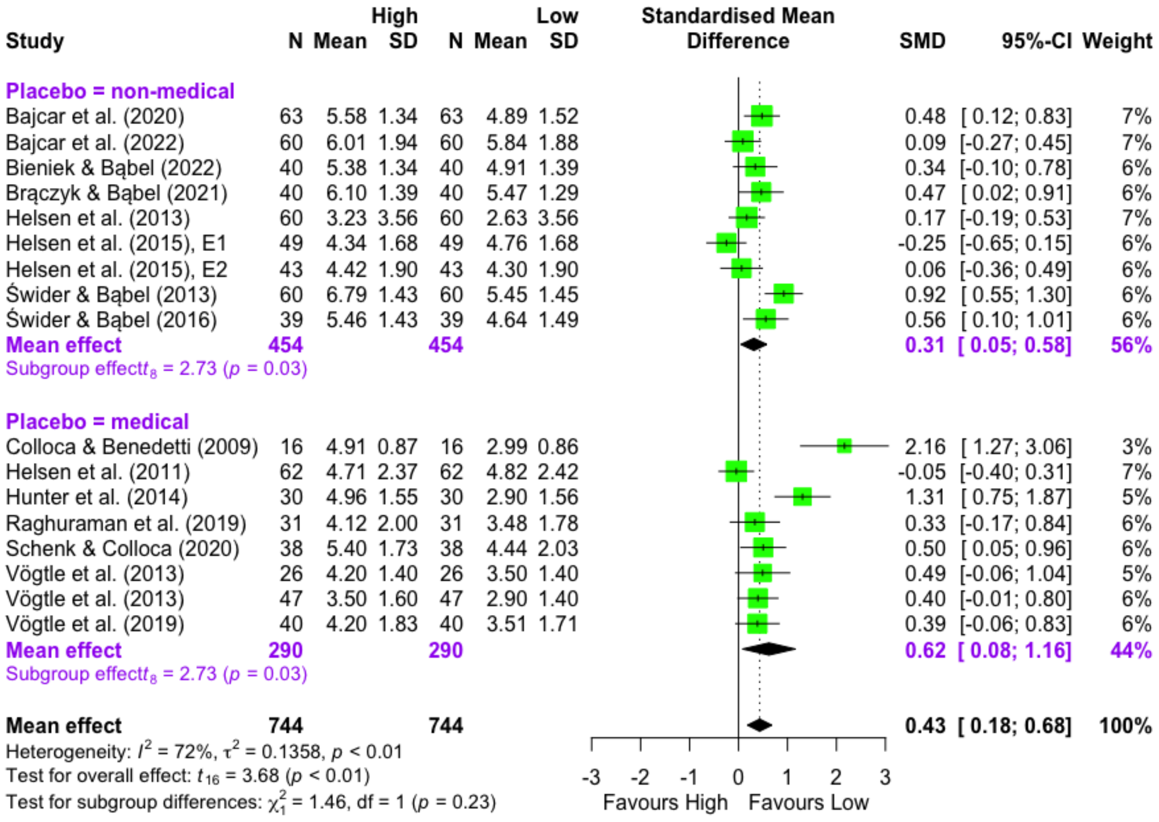

Supplementary Tables

Supplementary Table S1. The search strategy used for the PubMed database.

**1**	observational learning
**2**	social learning
**3**	social modeling
**4**	vicarious learning
**5**	social observation
**6**	#1 OR #2 OR #3 OR #4 OR #5
**7**	placebo analgesia
**8**	placebo hypoalgesia
**9**	nocebo hyperalgesia
**10**	placebo
**11**	nocebo
**12**	#7 OR #8 OR #9 OR #10 OR #11
**13**	#6 AND #12

Other databases used a similar strategy, modified where needed to suit the particular search engine

Supplementary Table S2. Inclusion and exclusion criteria according to the PICOS principle.

Category	Inclusion criteria	Exclusion criteria
**P**	healthy participants or pain patients enrolled in clinical trials	—
**I**	OL used to induce the placebo or nocebo effect in pain	no OL
	studies in which the model that is displaying pain behaviors is observed in-person or on a video recording, or a form of verbal modeling is used	
**C**	within-subject control	—
	between-subject control	
	no intervention or manipulation	
**O**	pain measured on any pain scale	—
	psycho-physiological correlates of pain	
	self-reported measures other than pain ratings (e.g., expectancy of pain)	
**S**	experimental studies	review articles, meta-analyses, conference proceedings
	randomized controlled trials	no full text available (publication in abstract form only)

P – population; I – intervention ; C – comparator; O – outcome; S – study type

Supplementary Table S3. Modifications to the Downs & Black risk of bias checklist.

Item number	Item	Reason for modifying/dropping the item
**4**	Were the settings of obtained comparator and outcome similar?	The settings for comparator and outcome were always similar (the studies were either within-subject or cross-over design, or the setting for the experimental and control groups were the same)
**11**	Were those subjects who were prepared to participate representative of the entire population from which they were recruited?	There was no way to assess the representativeness of the subjects in the context of the entire population, as data on dropouts during the selection process were not reported.
**14**	If any of the results of the study were not based on "data dredging"?	The results of studies included in our sample were unlikely to be based on data dredging, given the relatively small number of participants and the limited datasets from which the results were obtained
**18**	Were losses of patients to follow-up taken into account?	There is no follow-up period per se in experimental studies of this type; the item was changed to reflect patients who were approached but declined to take part (i.e., were enrolled but not evaluated). Also, participants who withdrew their participation were not analyzed at all.
**19**	Did the study have sufficient power to detect a clinically important effect where the probability value of a difference being due to chance is less than 5%?	Zero points were given when no power calculation was done or reviewers were unable to determine its existence from the publication; 1–6 points corresponded to the study having power of <75%, 76–80%, 81–85%, 86–90%, 91–95%, and >96% respectively.
